# Supplementary figures and images for: Genetic effects on life-history traits in the Glanville fritillary butterfly
Source: PeerJ. 2017 May 25;5:e3371. doi: 10.7717/peerj.3371 (PMC5446771; doi:10.7717/peerj.3371)

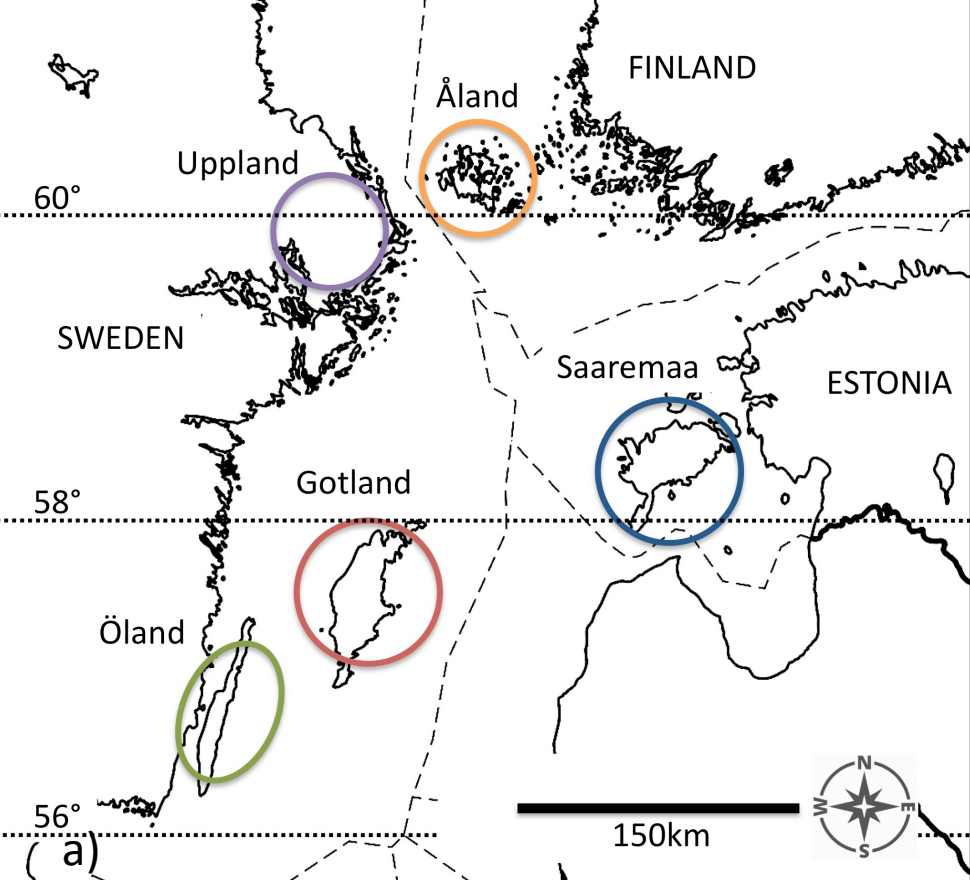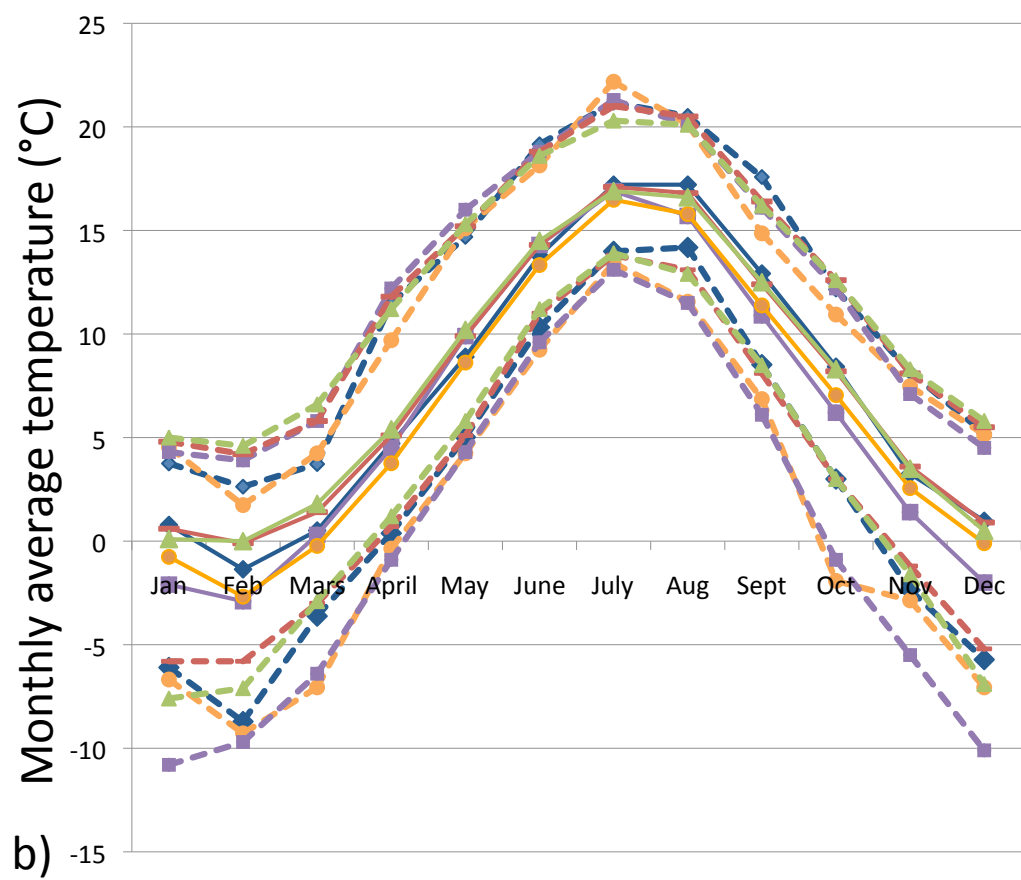

Supplement: Supplemental Information 1 — (A) Map of the Baltic Sea region with five populations of the Glanville fritillary: the Uppland coastal region, and Öland, Gotland, Saaremaa and Åland Islands. (B) Average monthly temperatures (full lines) and average monthly maximum and minimum temperatures (dashed) from 1992 to 2001 in Åland (orange), Uppland (purple), Öland (green), Gotland (red) and Saaremaa (blue). The map was created by A. Duplouy using a modified version of the image “Location map of the Baltic Sea” (https://commons.wikimedia.org/wiki/File:Baltic_Sea_location_map.svg, under the licence CC BY-SA 3.0, https://creativecommons.org/licenses/by-sa/3.0/) by NordNordWest/Wikipedia. The original image was cropped using Adobe Photoshop CS6 (Version: 13.06 × 64, http://www.adobe.com/products/photoshop.html). [file peerj-05-3371-s001.pdf]

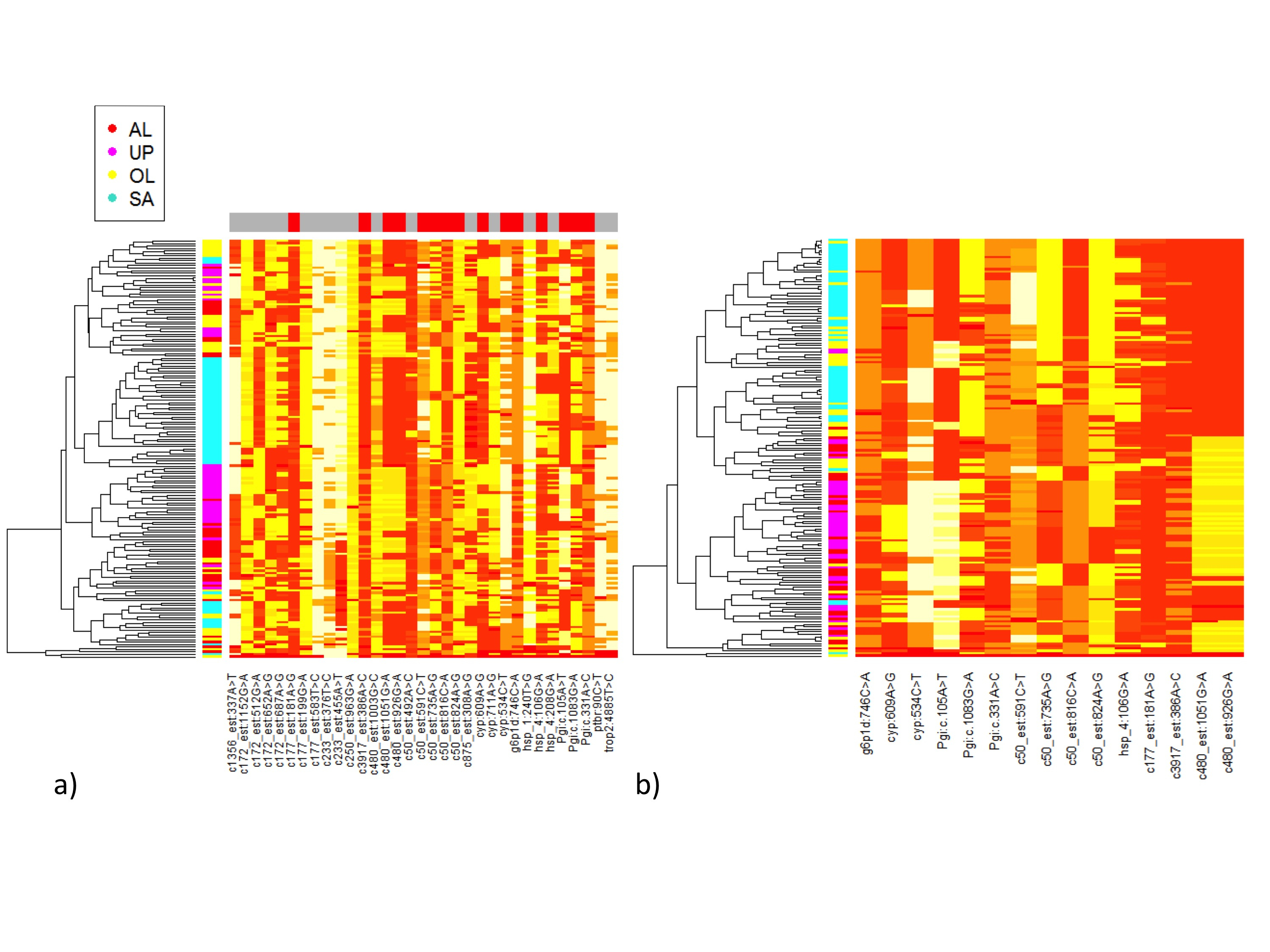

Supplement: Supplemental Information 2 — Dendograms on the Y-axes show the hierarchical clustering of all samples used in the study, color coded by population of origin (ÅL, UP, ÖL and SA, in red, magenta, yellow and turquoise, respectively). The SNPs appear on the X-axis. (a) The heatmap includes 33 SNPs. The red colour at the top coloured panel highlights the15 SNPs with significant allele frequencies differences between landscape type (northern fragmented, Åland and Uppland, versus southern continuous, Saaremaa and Öland). (b) The heatmap includes only the 15 SNPs with allele frequency differences between landscape types. Only samples collected in 2009 are included in this analysis. [file peerj-05-3371-s002.jpg]

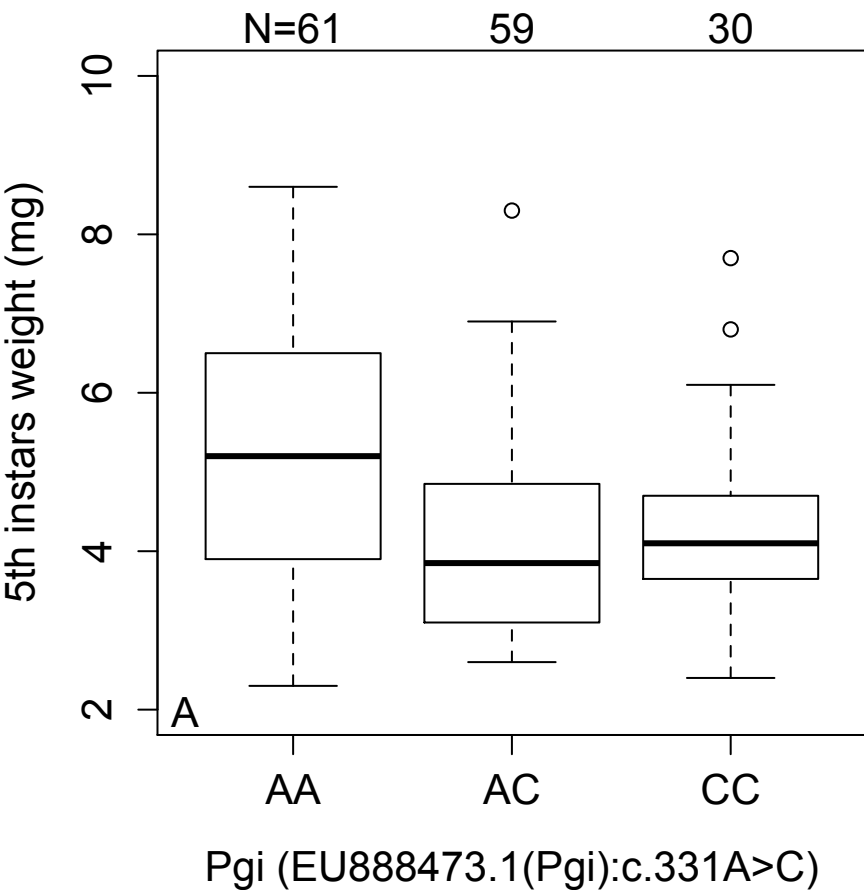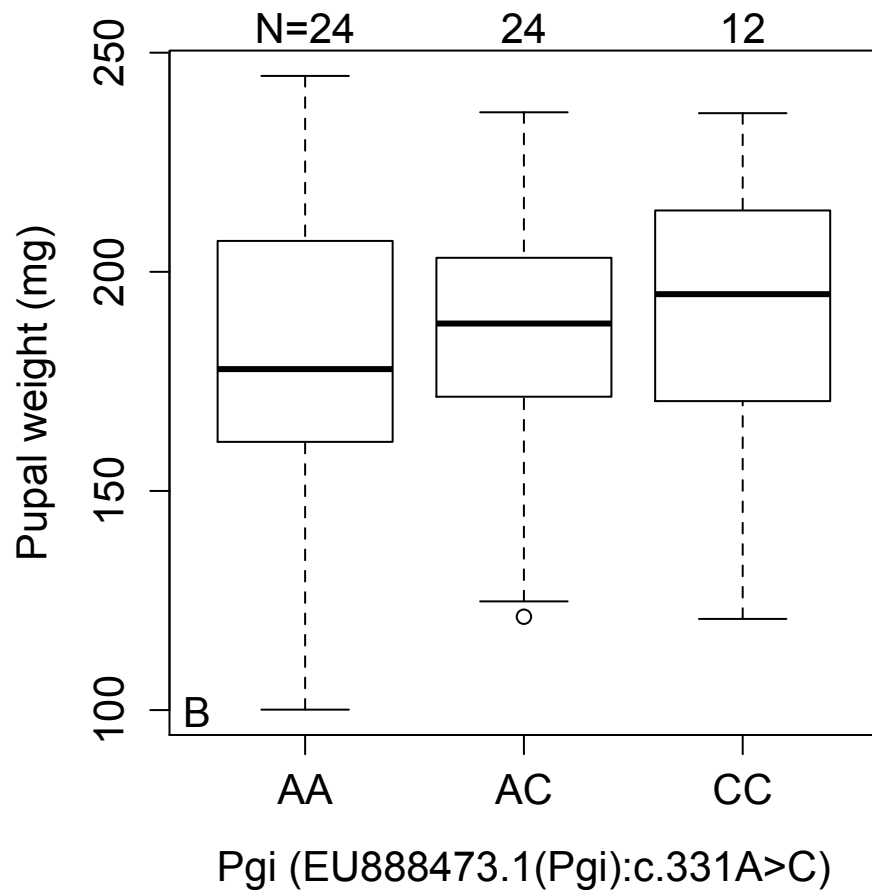

Supplement: Supplemental Information 3 — (A) The 5th instar larval weight (mg) for the different genotypes of samples collected in 2009. (B) The pupal weight (mg) for the different genotypes of samples collected in 2006. Sample size is given by the number above the bar. Heavy horizontal lines represent median values, boxes give interquartile ranges, whiskers and dots give the minimum and maximum, and outlier values, respectively. [file peerj-05-3371-s003.pdf]

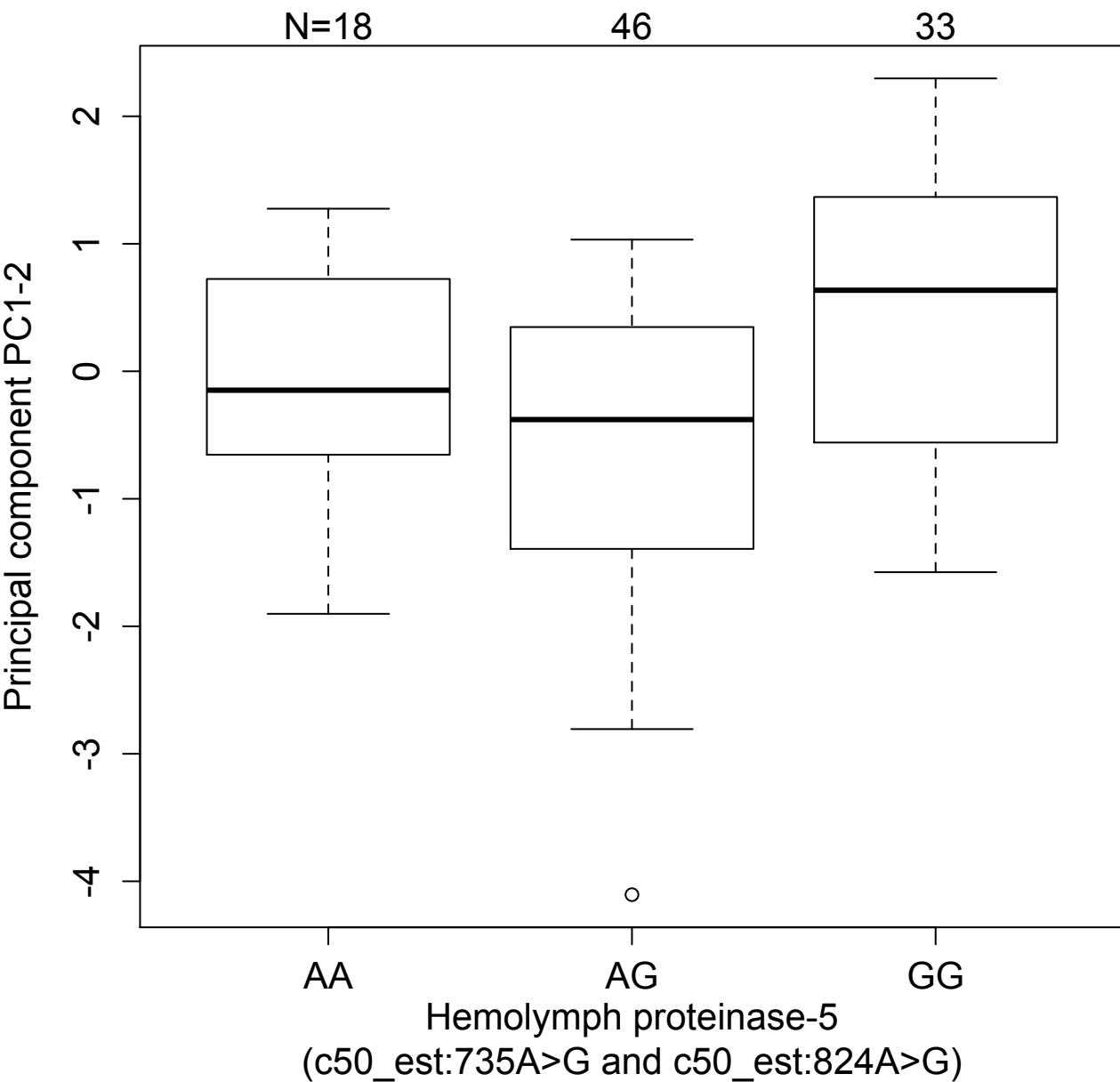

Supplement: Supplemental Information 4 — Sample size for each category is given by the number at the top of the graph. Heavy horizontal lines represent median values, boxes give interquartile ranges, whiskers give minimum and maximum values, dots represent outliers. [file peerj-05-3371-s004.pdf]

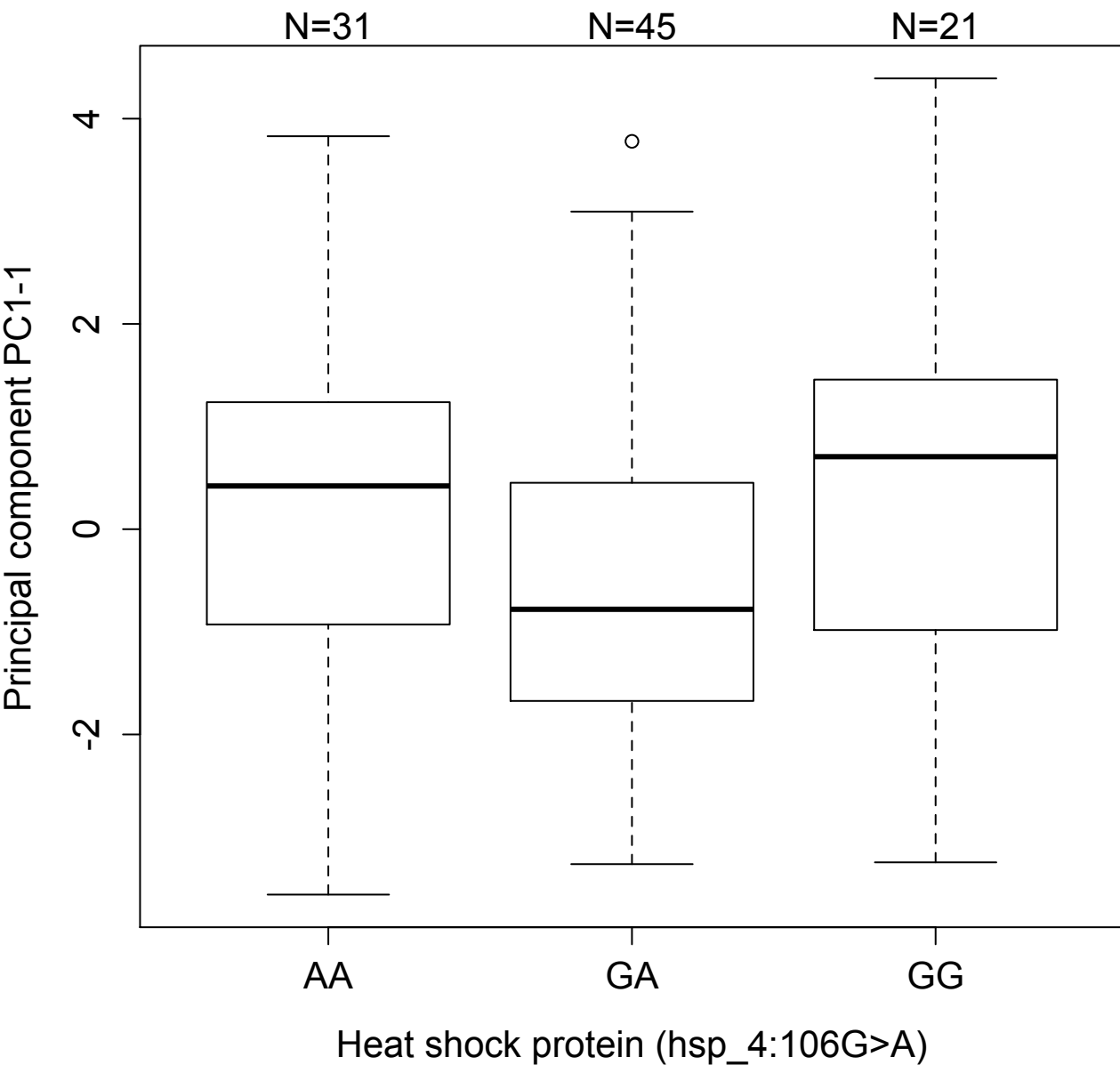

Supplement: Supplemental Information 5 — Sample size for each category is given by the number at the top of the graph. Heavy horizontal lines represent median values, boxes give interquartile ranges, whiskers give minimum and maximum values, dots represent outliers. [file peerj-05-3371-s005.pdf]

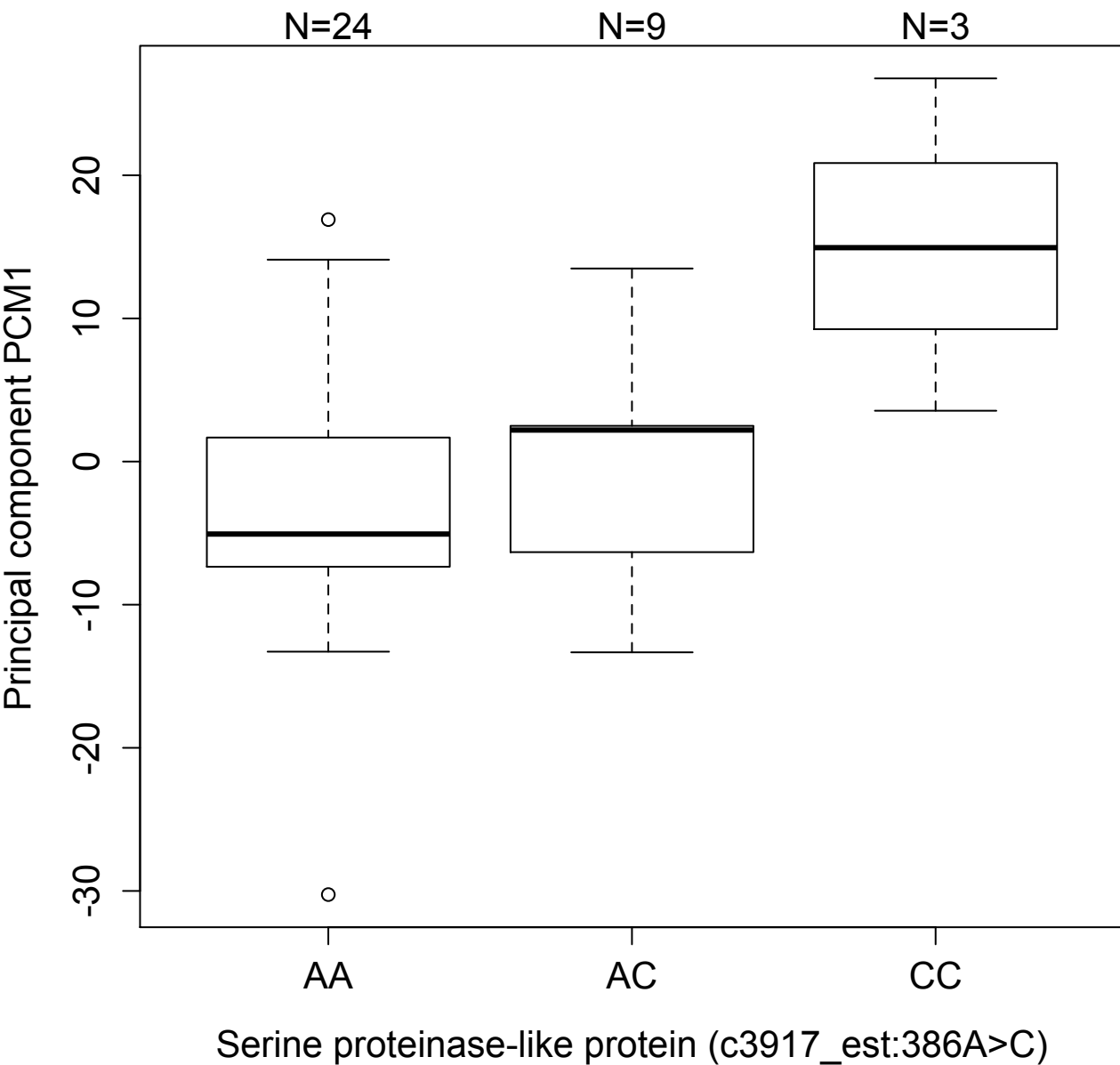

Supplement: Supplemental Information 6 — Sample size for each category is given by the number at the top of the graph. Heavy horizontal lines represent median values, boxes give interquartile ranges, whiskers give minimum and maximum values, dots represent outliers. [file peerj-05-3371-s006.pdf]

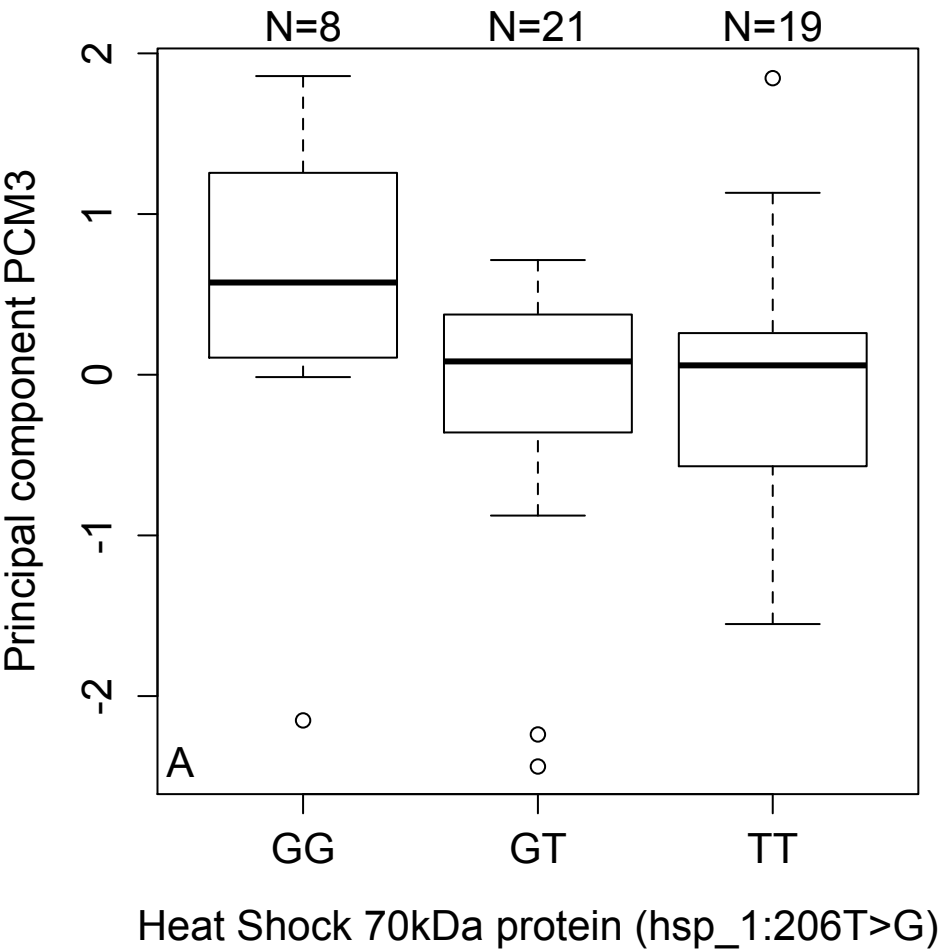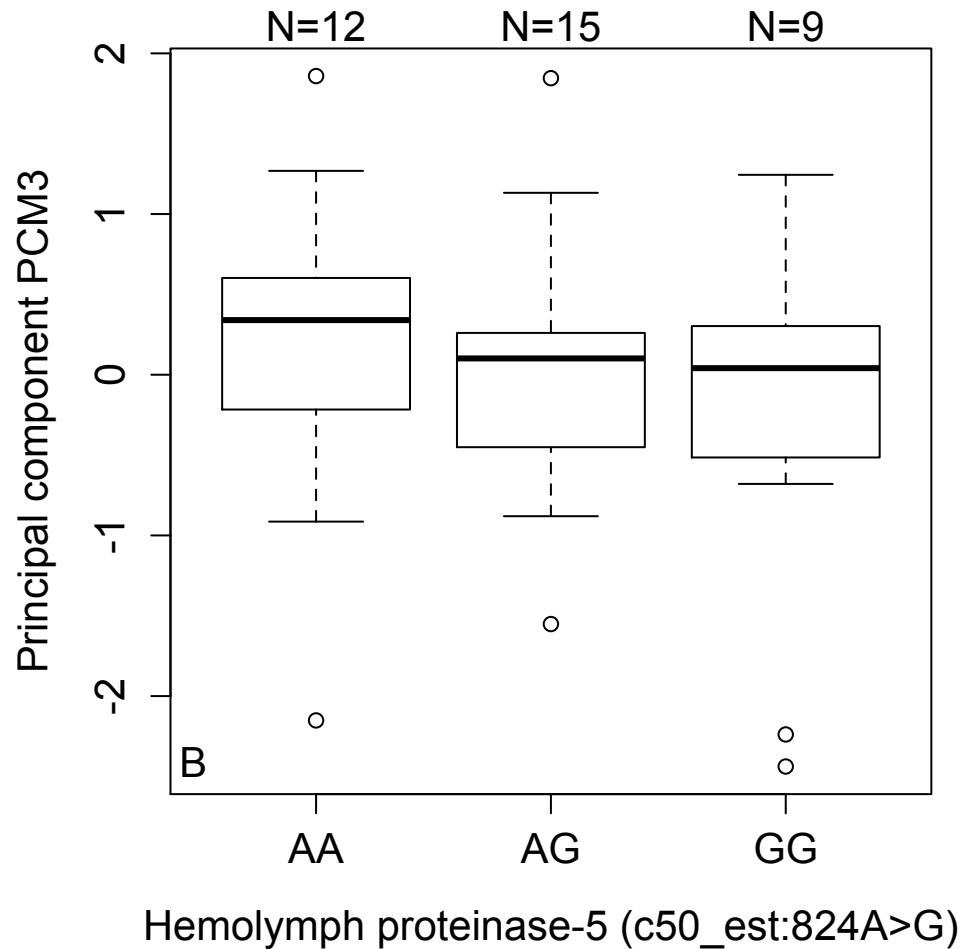

Supplement: Supplemental Information 7 — Sample size for each category is given by the number at the top of the graph. Heavy horizontal lines represent median values, boxes give interquartile ranges, whiskers give minimum and maximum values, dots represent outliers. [file peerj-05-3371-s007.pdf]
