# Supplementary material for: Genetic effects on life-history traits in the Glanville fritillary butterfly
Source: PeerJ. 2017 May 25;5:e3371. doi: 10.7717/peerj.3371 (PMC5446771; doi:10.7717/peerj.3371)
Supplement: Supplemental Information 8 — In the main experiment (2009) and the pilot experiment (2007), and number of individuals included in each PCA1 (larval and pupal traits only), PCAM (male adult traits only) and PCAF (female adult traits only). [file peerj-05-3371-s008.docx]

| **Environment** | **Northern fragmented** | | | | **Southern continuous** | | | |
| --- | --- | --- | --- | --- | --- | --- | --- | --- |
| **Main experiment 2009** | Åland (ÅL) | | Uppland (UP) | | Öland (ÖL) | | Saaremaa (SA) | |
| Sex | ♂ | ♀ | ♂ | ♀ | ♂ | ♀ | ♂ | ♀ |
| Samples (N=) | 21 | 14 | 26 | 18 | 22 | 17 | 27 | 31 |
| **Pilot experiment 2007** | Åland (ÅL) | | Uppland (UP) | | Gotland (GO) | | Saaremaa (SA) | |
| Sex | ♂ | ♀ | ♂ | ♀ | ♂ | ♀ | ♂ | ♀ |
| Samples (N=) | 16 | 13 | 19 | 28 | 6 | 9 | 16 | 15 |
| **PCA_1_ - Development** | Åland (ÅL) | | Uppland (UP) | | Öland (ÖL) | | Saaremaa (SA) | |
| Sex | ♂ | ♀ | ♂ | ♀ | ♂ | ♀ | ♂ | ♀ |
| Samples (N=) | 20 | 14 | 17 | 14 | 15 | 19 | 25 | 28 |
| **PCA_M_ -**  **Male traits** | Åland (ÅL) | | Uppland (UP) | | Öland (ÖL) | | Saaremaa (SA) | |
| Samples (N=) | 16 | - | 11 | - | 10 | - | 12 | - |
| **PCA_F_ -**  **Female traits** | Åland (ÅL) | | Uppland (UP) | | Öland (ÖL) | | Saaremaa (SA) | |
| Samples (N=) | - | 9 | - | 12 | - | 11 | - | 17 |
