# Supplementary material for: Genetic effects on life-history traits in the Glanville fritillary butterfly
Source: PeerJ. 2017 May 25;5:e3371. doi: 10.7717/peerj.3371 (PMC5446771; doi:10.7717/peerj.3371)
Supplement: Supplemental Information 9 — Values are Pearson correlative values (R) and asterisks denote significance at the 0.05 (*), 0.01 (**) and 0.001 (***) levels. The Bonferroni alpha values were corrected for 27, 15 and 91 correlations, respectively. [file peerj-05-3371-s009.docx]

| **Larval and pupal development traits (PCA_1_)** | | | | | | | | | | | | | | | | | | | | | | | | |
| --- | --- | --- | --- | --- | --- | --- | --- | --- | --- | --- | --- | --- | --- | --- | --- | --- | --- | --- | --- | --- | --- | --- | --- | --- |
|  | | 5^th^ weight | | | | 6^th^ weight | | | 7^th^ weight | | 5^th^ period | | | 6^th^ period | | | | 7^th^ period | | | | Pupal period | |  |
| 6^th^ weight | | 0.417 * | | | | - | | |  | |  | | |  | | | |  | | | |  | |  |
| 7^th^ weight | | 0.089 | | | | 0.236 | | | - | |  | | |  | | | |  | | | |  | |  |
| 5^th^ period | | -0.504 ** | | | | 0.085 | | | -0.052 | | - | | |  | | | |  | | | |  | |  |
| 6^th^ period | | -0.199 | | | | -0.333 | | | 0.445 * | | 0.243 | | | - | | | |  | | | |  | |  |
| 7^th^ period | | -0.088 | | | | -0.133 | | | -0.382 * | | 0.139 | | | 0.022 | | | | - | | | |  | |  |
| Pupal period | | 0.247 | | | | 0.161 | | | 0.142 | | -0.576 ** | | | -0.505 ** | | | | -0.646 *** | | | | - | |  |
| Pupal weight | | -0.006 | | | | -0.046 | | | 0.195 | | 0.165 | | | 0.427 * | | | | 0.634 *** | | | | -0.573 ** | |  |
| **Male adult traits (PCA_M_)** | | | | | | | | | | | | | | | | | | | | | | | | |
|  | | | Pupal weight | | | | | Distance | | | Proba to fly ^T^ | | | | | Nbr of matings | | | | Survival | | | | |
| Distance | | | 0.059 | | | | | - | | |  | | | | |  | | | |  | | | | |
| Proba to fly ^T^ | | | 0.095 | | | | | 0.215 | | | - | | | | |  | | | |  | | | | |
| Nbr of matings | | | -0.268 | | | | | -0.043 | | | 0.101 | | | | | - | | | |  | | | | |
| Survival | | | 0.236 | | | | | 0.242 | | | 0.021 | | | | | 0.099 | | | | - | | | | |
| Age at 1^st^ mating | | | -0.228 | | | | | 0.100 | | | -0.051 | | | | | -0.027 | | | | 0.306 | | | | |
| **Female adult traits (PCA_F_)** | | | | | | | | | | | | | | | | | | | | | | | | |
|  | Pupal weight | | | Distance | Proba to fly ^T^ | | Plant pref ^T^ | | Nbr of matings | Age at 1^st^ ovip | | Survival | Total clutch | | Total larvae | | Total eggs | | Hatch rate ^T^ | | Age 1^st^ mating | | Size 1^st^ clutch | |
| Distance | 0.320 ** | | | - |  | |  | |  |  | |  |  | |  | |  | |  | |  | |  | |
| Proba to fly^T^ | 0.080 | | | 0.190 | - | |  | |  |  | |  |  | |  | |  | |  | |  | |  | |
| Plant pref ^T^ | -0.158 | | | 0.007 | -0.118 | | - | |  |  | |  |  | |  | |  | |  | |  | |  | |
| Nbr of matings | 0.003 | | | -0.026 | -0.103 | | 0.007 | | - |  | |  |  | |  | |  | |  | |  | |  | |
| Age at 1^st^ oviposition | 0.133 | | | 0.130 | 0.162 | | 0.176 | | 0.041 | - | |  |  | |  | |  | |  | |  | |  | |
| Survival | -0.036 | | | 0.225 * | -0.053 | | -0.113 | | 0.160 | 0.252 * | | - |  | |  | |  | |  | |  | |  | |
| Total clutch | 0.005 | | | 0.163 | -0.075 | | -0.156 | | 0.237* | -0.151 | | 0.687 *** | - | |  | |  | |  | |  | |  | |
| Total larvae | 0.216 * | | | 0.282 ** | 0.029 | | -0.191 | | 0.174 | -0.005 | | 0.728 *** | 0.808*** | | - | |  | |  | |  | |  | |
| Total eggs | 0.216 * | | | 0.230 * | -0.013 | | -0.182 | | 0.224* | -0.002 | | 0.768 *** | 0.862*** | | 0.979 *** | | **-** | |  | |  | |  | |
| Hatch rate^T^ | 0.331 ** | | | 0.300 ** | 0.277** | | -0.36 *** | | -0.084 | 0.005 | | 0.243 * | 0.187 | | 0.511 *** | | 0.372 *** | | **-** | |  | |  | |
| Age 1^st^ mating | -0.202 | | | -0.168 | 0.013 | | 0.177 | | -0.111 | -0.032 | | -0.014 | -0.058 | | -0.059 | | -0.023 | | -0.329** | | **-** | |  | |
| 1^st^ clutch size | 0.242 * | | | 0.052 | 0.294 ** | | -0.130 | | -0.109 | 0.464 *** | | 0.097 | -0.27 * | | 0.142 | | 0.102 | | 0.280 ** | | -0.115 | | **-** | |
| HR 1^st^ clutch^T^ | 0.265 * | | | 0.222 * | 0.253 * | | -0.32 ** | | -0.293 ** | 0.055 | | 0.161 | -0.034 | | 0.349 *** | | 0.212 * | | 0.825 *** | | -0.215 * | | 0.498 *** | |
