# Supplementary material for: Genetic effects on life-history traits in the Glanville fritillary butterfly
Source: PeerJ. 2017 May 25;5:e3371. doi: 10.7717/peerj.3371 (PMC5446771; doi:10.7717/peerj.3371)
Supplement: Supplemental Information 10 — Highest values from each PCA appear in bold for easier visualization of the results. [file peerj-05-3371-s010.docx]

|  | | **PC1** | | **PC2** | | **PC3** | **PC4** |
| --- | --- | --- | --- | --- | --- | --- | --- |
| **Eigen value** | | 2.87 | | 1.59 | | 1.16 | 1.14 |
| **Cumulative proportion of variance** | | 0.36 | | 0.56 | | 0.74 | 0.88 |
| ***Larval and pupal development*** | | | | | | | |
| 5^th^ instar weight | -0.308 | | -0.235 | | **-0.538** | | 0.146 |
| 6^th^ instar weight | -0.186 | | -0.214 | | **-0.430** | | **-0.669** |
| 7^th^ instar weight | -0.034 | | **-0.733** | | 0.132 | | -0.080 |
| 5^th^ instar period | 0.359 | | 0.094 | | 0.216 | | **-0.647** |
| 6^th^ instar period | 0.344 | | **-0.476** | | 0.271 | | 0.226 |
| 7^th^ instar period | **0.403** | | 0.237 | | **-0.475** | | 0.162 |
| Pupal weight | **0.409** | | -0.263 | | -0.386 | | 0.141 |
| Pupal period | **-0.541** | | -0.010 | | 0.117 | | 0.094 |
