# Supplementary material for: Genetic effects on life-history traits in the Glanville fritillary butterfly
Source: PeerJ. 2017 May 25;5:e3371. doi: 10.7717/peerj.3371 (PMC5446771; doi:10.7717/peerj.3371)
Supplement: Supplemental Information 11 — Highest values from each PCA appear in bold for easier visualization of the results. [file peerj-05-3371-s011.docx]

|  | | **PC1** | | **PC2** | **PC3** | |  |  |
| --- | --- | --- | --- | --- | --- | --- | --- | --- |
| **Eigen value** | | 1.22 | | 1.16 | 1.07 | |  |  |
| **Cumulative proportion of variance** | | 0.25 | | 0.47 | 0.66 | |  |  |
| ***Male adult traits*** | | | | | | | | |
| Pupal weight | -0.285 | | **-0.679** | | | -0.106 | |  |
| Distance | **-0.552** | | -0.023 | | | 0.213 | |  |
| Probability to fly ^T^ | -0.282 | | -0.129 | | | **0.687** | |  |
| Number of matings | 0.036 | | 0.483 | | | **0.525** | |  |
| Survival | **-0.639** | | 0.117 | | | -0.189 | |  |
| Age at 1^st^ Mating | -0.354 | | **0.525** | | | **-0.400** | |  |
