# Supplementary material for: Genetic effects on life-history traits in the Glanville fritillary butterfly
Source: PeerJ. 2017 May 25;5:e3371. doi: 10.7717/peerj.3371 (PMC5446771; doi:10.7717/peerj.3371)
Supplement: Supplemental Information 12 — Highest values from each PCA appear in bold for easier visualization of the results. [file peerj-05-3371-s012.docx]

|  | **PC1** | **PC2** | **PC3** |
| --- | --- | --- | --- |
| **Eigen value** | 2.02 | 1.60 | 1.22 |
| **Cumulative proportion of variance** | 0.29 | 0.48 | 0.58 |
| ***Female adult traits*** | | | |
| Pupal weight | 0.165 | 0.242 | 0.001 |
| Distance | 0.205 | 0.119 | -0.107 |
| Probability to fly ^T^ | 0.076 | 0.291 | -0.121 |
| Host-plant preference | -0.174 | -0.137 | **-0.419** |
| Number of matings | 0.066 | -0.257 | -0.130 |
| Age at 1st ovipositon | 0.049 | 0.176 | **-0.684** |
| Survival | **0.369** | -0.220 | -0.247 |
| Total numer of clutches | **0.356** | **-0.378** | 0.074 |
| Total number of caterpillars | **0.461** | -0.156 | -0.021 |
| Size of 1^st^ clutch | 0.131 | **0.397** | **-0.352** |
| Total number of eggs | **0.442** | -0.232 | -0.059 |
| Mean hatch rate ^T^ | **0.339** | **0.311** | 0.237 |
| Hatch rate 1st clutch ^T^ | 0.266 | **0.418** | 0.165 |
| Age at 1st mating | -0.115 | -0.168 | -0.190 |
