# Supplementary material for: Genetic effects on life-history traits in the Glanville fritillary butterfly
Source: PeerJ. 2017 May 25;5:e3371. doi: 10.7717/peerj.3371 (PMC5446771; doi:10.7717/peerj.3371)
Supplement: Supplemental Information 13 — Each Pgi locus was genotyped twice. SNPs tagged with “(f)” failed our quality criteria, while SNPs tagged with “(*)” were genotyped for the 2007 pilot study. HWE, Hardy Weinberg Equilibrium; MAF, Minimum Allele Frequency; Gen, Genotyping; Homoz, Homozygote. Aland (AL) and Uppland (UP) populations evolve in northern fragmented environments, while Gotland (GO), Oland (OL) and Saaremaa (SA) populations evolve in southern continuous environments. [file peerj-05-3371-s013.docx]

| Environments | **Northern fragmented** | | | | | | | | | | **Southern continuous** | | | | | | | |
| --- | --- | --- | --- | --- | --- | --- | --- | --- | --- | --- | --- | --- | --- | --- | --- | --- | --- | --- |
| Populations | **ÅL** | | | | | **UP** | | | | | **ÖL (GO for 2007 data)** | | | | **SA** | | | |
| SNP name | Minor Allele | MAF | | | Failed quality criterion | Minor Allele | MAF | | | Failed quality criterion | Minor Allele | | MAF | Failed quality criterion | Minor Allele | | MAF | Failed quality criterion |
| ***Glycolytic enzyme Phosphoglucose isomerase – Pgi*** | | | | | | | | | | | | | | | | | | |
| EU888473.1(Pgi):c.105A>T | T | | | 0.4714 | - | A | | 0.2857 | | - | | T | 0.2571 | - | | T | 0.0083 | MAF |
| EU888473.1(Pgi):c.105A>T | T | | | 0.4722 | - | A | | 0.2738 | | - | | T | 0.2571 | - | | T | 0.0085 | MAF |
| EU888473.1(Pgi):c.1083G>A | A | | | 0.4167 | - | A | | 0.3462 | | - | | A | 0.2647 | - | | A | 0.1610 | - |
| EU888473.1(Pgi):c.1083G>A | A | | | 0.3871 | - | A | | 0.3553 | | - | | A | 0.2571 | - | | A | 0.1667 | - |
| EU888473.1(Pgi):c.1083G>A^(f)(*)^ | A | | | 0.362 | HWE | A | | 0.347 | | HWE | | A | 0.300 | HWE | | A | 0.355 | HWE |
| EU888473.1(Pgi):c.331A>C | C | | | 0.2000 | - | C | | 0.2500 | | - | | A | 0.3286 | - | | C | 0.4417 | - |
| EU888473.1(Pgi):c.331A>C | C | | | 0.1944 | - | C | | 0.2561 | | - | | A | 0.3286 | - | | C | 0.4417 | - |
| EU888473.1(Pgi):c.331A>C ^(*)^ | C | | | 0.241 | - | C | | 0.266 | | HWE | | A | 0.233 | HWE | | A | 0.452 | - |
| ***Hemolymph proteinase 5*** | | | | | | | | | | | | | | | | | | |
| c50_est:591C>T | T | | 0.3429 | | - | T | | | 0.2927 | - | | T | 0.3529 | HWE | | C | 0.4492 | - |
| c50_est:735A>G | G | | 0.4857 | | - | G | | | 0.4762 | - | | A | 0.2571 | - | | A | 0.1186 | HWE |
| c50_est:816C>A | A | | 0.4861 | | - | A | | | 0.4762 | - | | C | 0.3429 | - | | C | 0.1333 | - |
| c50_est:492A>G | G | | 0.1250 | | - | G | | | 0.1667 | - | | G | 0.2222 | - | | G | 0.2000 | - |
| c50_est:824A>G | G | | 0.4861 | | - | G | | | 0.4762 | - | | A | 0.2571 | - | | A | 0.1017 | HWE |
| ***Serpin 1*** | | | | | | | | | | | | | | | | | | |
| c172_est:1152G>A | A | | 0.4857 | | - | A | | | 0.0976 | MAF | | A | 0.1286 | - | | A | 0.2881 | - |
| c172_est:512G>A | A | | 0.3429 | | - | A | | | 0.2143 | - | | A | 0.3143 | - | | G | 0.3333 | - |
| c172_est:687A>G | G | | 0.4722 | | - | A | | | 0.2805 | - | | A | 0.2857 | - | | A | 0.3136 | - |
| c172_est:652A>G | G | | 0.3889 | | - | G | | | 0.3415 | HW | | G | 0.3677 | - | | A | 0.3190 | - |
| step3_c172_622.4706 ^(f)^ | - | | - | | Gen | - | | | - | Gen | | - | - | Gen | | - | - | Gen |
| ***Heat shock 70 kDa protein*** | | | | | | | | | | | | | | | | | | |
| hsp_1:206T>G | G | | 0.4849 | | - | G | | | 0.3902 | - | | T | 0.4118 | - | | G | 0.0750 | - |
| hsp_1:134C>T ^(f)^ | T | | 0.1857 | | - | T | | | 0.1667 | - | | C | 1 | Homoz | | T | 0.0083 | MAF |
| hsp_2:100G>A ^(f)^ | A | | 0.1714 | | - | A | | | 0.0595 | - | | A | 0.0143 | MAF | | A | 0.0167 | MAF |
| hsp_4:106G>A | G | | 0.4857 | | - | G | | | 0.2619 | - | | A | 0.4429 | - | | A | 0.4000 | HWE |
| hsp_4:268A>G | G | | 0.4857 | | - | G | | | 0.2619 | - | | A | 0.4571 | - | | G | 0.4417 | - |
| hsp3:71T ^(f)^ | T | | 1 | | Homoz | T | | | 1 | Homoz | | T | 1 | Homoz | | T | 1 | Homoz |
| ***Vitellin-degrading protease precursor*** | | | | | | | | | | | | | | | | | | |
| c177_est:181A>G | G | | 0.0556 | | - | G | | | 0.0119 | MAF | | G | 0.3857 | HWE | | G | 0.2417 | - |
| c177_est:199G>A | A | | 0.4583 | | - | A | | | 0.4512 | - | | A | 0.1912 | - | | G | 1 | Homoz |
| c177_est:583T>C | C | | 0.0972 | | - | C | | | 0.0357 | MAF | | T | 1 | Homoz | | C | 0.1000 | - |
| ***Chymotrypsinogen-like protein*** | | | | | | | | | | | | | | | | | | |
| c1356_est:337A>T | T | | 0.4142 | | - | A | | | 0.2561 | - | | A | 0.4571 | - | | A | 0.0417 | MAF |
| ***Prophenoloxidase-activating proteinase-3*** | | | | | | | | | | | | | | | | | | |
| step3_c233_670.4718 ^(f)^ | - | | - | | Gen | - | | | - | Gen | | - | - | Gen | | - | - | Gen |
| c233_est:376T>C | C | | 0.4167 | | - | C | | | 0.2143 | - | | C | 0.2500 | - | | C | 0.1525 | - |
| c233_est:455A>T | T | | 0.4286 | | - | A | | | 0.2250 | - | | A | 0.4394 | - | | A | 0.2966 | HWE |
| c233_est:581T>A ^(f)^ | A | | 0.0417 | | MAF | A | | | 0.0238 | MAF | | A | 0.0429 | MAF | | T | 1 | Homoz |
| ***Serine proteinase-like protein 1*** | | | | | | | | | | | | | | | | | | |
| c3917_est:386A>C | C | | 0.2429 | | - | C | | | 0.1905 | - | | C | 0.1429 | - | | A | 1 | Homoz |
| ***Endocuticle structural glycoprotein – SgAbd-8*** | | | | | | | | | | | | | | | | | | |
| c480_est:1051G>A | A | | 0.4722 | | - | A | | | 0.4500 | - | | G | 0.1714 | - | | G | 0.0085 | MAF |
| c480_est:926G>A | A | | 0.4722 | | - | A | | | 0.4512 | - | | G | 0.1714 | - | | G | 0.0085 | MAF |
| c480_est:1003G>C | C | | 0.1806 | | - | C | | | 0.3415 | - | | C | 0.3143 | - | | G | 0.3390 | - |
| step3_c807_799.4711 ^(f)^ | - | | - | | Gen | - | | | - | Gen | | - | - | Gen | | - | - | Gen |
| ***Cuticular protein*** | | | | | | | | | | | | | | | | | | |
| c2634_est:308C>T ^(f)^ | T | | 0.4429 | | - | T | | | 0.2195 | - | | T | 0.0441 | MAF | | C | 1 | Homoz |
| ***Kazal-type proteinase inhibitor*** | | | | | | | | | | | | | | | | | | |
| c875_est:308A>G | G | | 0.3889 | | - | A | | | 0.4375 | - | | A | 0.4000 | - | | G | 0.1042 | - |
| ***Prophenoloxidase-activating proteinase-1*** | | | | | | | | | | | | | | | | | | |
| c250_est:963G>A | A | | 0.2429 | | - | A | | | 0.3625 | HW | | A | 0.3889 | HW | | A | 0.2034 | - |
| ***Trypsin-like protease*** | | | | | | | | | | | | | | | | | | |
| c2605_est:563T>C ^(f)^ | C | | 0.3889 | | HW | C | | | 0.3691 | HW | | C | 0.2941 | HW | | C | 0.4083 | HW |
| step3_c2605_766.4701 ^(f)^ | - | | - | | Gen | - | | | - | Gen | | - | - | Gen | | - | - | Gen |
| ***Cytochrome P450*** | | | | | | | | | | | | | | | | | | |
| cyp:263A>G | G | | 0.4286 | | - | A | | | 0.2857 | - | | G | 0.0571 | - | | G | 0.2373 | - |
| cyp:365A>G | G | | 0.4286 | | - | A | | | 0.2738 | - | | G | 0.3971 | - | | A | 0.1271 | HW |
| cyp:188C>T | T | | 0.4286 | | - | C | | | 0.2976 | - | | T | 0.0429 | MAF | | T | 0.2328 | - |
| ***Glucose-6-phosphate 1-dehydrogenase*** | | | | | | | | | | | | | | | | | | |
| g6p1d:113T>G ^(f)^ | G | | 0.1714 | | - | G | | | 0.0732 | - | | T | 1 | Homoz | | T | 1 | Homoz |
| g6p1d:239C>T | C | | 0.2286 | | - | C | | | 0.1786 | - | | A | 0.4265 | - | | A | 0.0250 | MAF |
| ***Peripheral-type benzodiazepine receptor*** | | | | | | | | | | | | | | | | | | |
| ptbr:140C>T | T | | 0.4143 | | - | C | | | 0.3049 | - | | C | 0.3088 | - | | C | 0.3793 | HW |
| ***Succinate dehydrogenase complex subunit D – SDHD*** | | | | | | | | | | | | | | | | | | |
| sdhd:149T>C ^(f)^ | C | | 0.1571 | | - | C | | | 0.0357 | MAF | | C | 0.0441 | MAF | | C | 0.0417 | MAF |
| ***Troponin-T*** | | | | | | | | | | | | | | | | | | |
| trop2:100T>C | C | | 0.3429 | | - | C | | | 0.2381 | HW | | C | 0.3571 | - | | C | 0.0678 | - |
| trop1:95T ^(f)^ | T | | 1 | | Homoz | T | | | 1 | Homoz | | T | 1 | Homoz | | T | 1 | Homoz |
| ***Flightin – Fln*** | | | | | | | | | | | | | | | | | | |
| fln:113G>T ^(f)^ | T | | 0.2571 | | - | T | | | 0.4524 | HW | | T | 0.3971 | HW | | T | 0.2034 | HW |
| ***C-Jun N-terminal protein kinase1*** | | | | | | | | | | | | | | | | | | |
| jnk1:183T ^(f)^ | T | | 1 | | Homoz | T | | | 1 | Homoz | | T | 1 | Homoz | | T | 1 | Homoz |
| ***Triosephosphate isomerase*** | | | | | | | | | | | | | | | | | | |
| trii:261A>G ^(f)^ | - | | - | | Gen | - | | | - | Gen | | - | - | Gen | | - | - | Gen |
