# Supplementary material for: Genetic effects on life-history traits in the Glanville fritillary butterfly
Source: PeerJ. 2017 May 25;5:e3371. doi: 10.7717/peerj.3371 (PMC5446771; doi:10.7717/peerj.3371)
Supplement: Supplemental Information 14 — Models of inheritance are given within brackets as Dominant, Recessive or Additive. Both main effects and interactions are reported. “NS” stands for non-significant. [file peerj-05-3371-s014.docx]

| **Trait** | **Models** | **Explanatory variables** | | | |
| --- | --- | --- | --- | --- | --- |
| ***PCA_1_ - Larval and pupal traits*** | | **Environment** | **Sex** | | **SNP (model of inheritance)** |
| Larval development (PC_1-1_) | PCA+ lme + FDR | NS | df>90,  *p*-value<1.52e-8 | | *hsp_1:106G>A*, df=92, t=-3.15, *p*-value=2.18e-3 |
| Larval development (PC_1-1_) | PCA+ lme + FDR | NS | df>90,  *p*-value<1.52e-8 | | All other 32 SNPs: NS |
| Larval development (PC_1-2_) | PCA+ lme + FDR | NS | NS | | *c50_est:735A>G,* df=92,  t=-3.36, *p*-value*=*1.15e-3 (Over-dominant) |
| Larval development (PC_1-2_) | PCA+ lme + FDR | NS | NS | | *c50_est:824A>G,* df=93,  t=-3.34, *p*-value=1.21e-3 (Over-dominant) |
| Larval development (PC_1-2_) | PCA+ lme + FDR | NS | NS | | Interaction environment by genotype, 2SNPS in *SgAbd-8*, In FL: G>A, df=91, t=-3.14,  *p*-values<2.26e-3 (Dominant) |
| Larval development (PC_1-2_) | PCA+ lme + FDR | NS | NS | | All other 30 SNPs: NS |
| Larval development (PC_1-3_) | PCA+ lme + FDR | NS | df>90,  *p*-value<3.1e-5 | | *(Pgi):c.331A>C,* df=145, t=3.64, *p*-value=3.84e-4 (Dominant) |
| Larval development (PC_1-3_) | PCA+ lme + FDR | NS | df>90,  *p*-value<3.1e-5 | | All other 32 SNPs: NS |
| Larval development (PC_1-4_) | PCA+ lme + FDR | NS | NS | | All 33 SNPs: NS |
| Larval time (days)  (2007 pilot study) | lme + FDR | NS | df=37,  *p*-value<5.80e-6 | | Both genotyped *Pgi* SNPs: NS |
| Pupal weight (mg)  (2007 pilot study) | lme + FDR | NS | df=37,  *p*-value=1.01e-7 | | *(Pgi):c.331A>C*, df=37, t=3.15, *p*-value=0.0032 (Dominant) |
| Pupal weight (mg)  (2007 pilot study) | lme + FDR | NS | df=37,  *p*-value=1.01e-7 | | Second *Pgi* SNP: NS |
| ***PCA_M_ - Adult male traits*** | | **Environment** | | **SNP (model of inheritance)** | |
| PC_M1_ | PCA+ lme + FDR | NS | | *c3917_est:386A>C*, df=32, *p*-value=1.64e-3 (Recessive) | |
| PC_M1_ | PCA+ lme + FDR | NS | | All other 32 SNPs: NS | |
| PC_M2_ | PCA+ lme + FDR | NS | | c480_est:1003G>C, df=43, *p*-value=2.15e-3 (Dominant) | |
| PC_M2_ | PCA+ lme + FDR | NS | | All other 32 SNPs: NS | |
| PC_M3_ | PCA+ lme + FDR | NS | | *c480_est:926G>A*, df=31, *p*-value*=*8.56e-4 (Dominant) | |
| PC_M3_ | PCA+ lme + FDR | NS | | *c480_est:1051G>A*, df=31, *p*-value*=*8.56e-4 (Dominant) | |
|  |  |  | | Interaction environment by genotype, 2 SNPS in *SgAbd-8*, df=31, t=-3.39,  *p*-values=1.87e-3 (over-dominant) | |
| PC_M3_ | PCA+ lme + FDR | NS | | *c50_est:824A>G*, df=31 , *p*-value=2.35e-3 (Additive) | |
| PC_M3_ | PCA+ lme + FDR | NS | | *hsp_1:206T>G*, df=42, *p*-value=1.33e-3 (Recessive) | |
| PC_M3_ | PCA+ lme + FDR | NS | | All other 29 SNPs: NS | |
| ***PCA_F_ - Adult female traits*** | | **Environment** | | **SNP (model of inheritance)** | |
| PC_F1_ | PCA+ lme + FDR | NS | | All 33 SNPs: NS | |
| PC_F2_ | PCA+ lme + FDR | NS | | All 33 SNPs: NS | |
| PC_F3_ | PCA+ lme + FDR | NS | | All 33 SNPs: NS | |
