# Supplementary material for: Genetic effects on life-history traits in the Glanville fritillary butterfly
Source: PeerJ. 2017 May 25;5:e3371. doi: 10.7717/peerj.3371 (PMC5446771; doi:10.7717/peerj.3371)
Supplement: Supplemental Information 15 — The minimum allele frequency for each SNP is shown in bold to ease visualization of the results. Allele frequencies differences between environments were calculated using directed permutation tests (1e+6 random permutations). The p-values give the posterior probability of the hypothesis that there is no difference between the environment types. [file peerj-05-3371-s015.docx]

| **Gene annotation** | **SNP name** | **Amino acid change** | **Allele frequencies** | | | | | | | **Permutation (*p*-value)** |
| --- | --- | --- | --- | --- | --- | --- | --- | --- | --- | --- |
|  |  |  | **ÅL** | | **UP** | | **ÖL/GO** | | **SA** |  |
| *Glycolytic enzyme phosphoglucose isomerase*  *(Pgi)* | *EU888473.1(Pgi):c.105A>T* | Non-Synonymous | A:0.53  **T:0.47** | | **A:0.29**  T:0.71 | | A:0.74  **T:0.26** | | A:0.992  **T:0.008** | < 10^-6 |
|  | *EU888473.1(Pgi):c.331A>C* | Non-Synonymous | A:0.80  **C:0.20** | | A:0.75  **C:0.25** | | **A:0.33**  C:0.67 | | **A:0.44**  C:0.56 | < 10^-6 |
|  | *EU888473.1(Pgi):c.331A>C** | Non-Synonymous* | A:0.76  **C:0.24** | | A:0.73  **C:0.27** | | **A:0.23**  C:0.77 | | **A:0.45**  C:0.55 | < 10^-6 |
|  | *EU888473.1(Pgi):c.1083G>A* | Synonymous | G:0.58  **A:0.42** | | G:0.65  **A:0.35** | | G:0.74  **A:0.26** | | G:0.84  **A:0.16** | < 10^-6 |
| *Hemolymph proteinase-5* | *c50_est:735A>G* | Synonymous | A:0.51  **G:0.49** | | A:0.52  **G:0.48** | | **A:0.26**  G:0.74 | | **A:0.12**  G:0.88 | < 10^-6 |
|  | *c50_est:591C>T* | Synonymous | C:0.66  **T:0.34** | | C:0.71  **T:0.29** | | C:0.65  **T:0.35** | | **C:0.45**  T:0.55 | < 10^-6 |
|  | *c50_est:816C>A* | Synonymous | C:0.51  **A:0.49** | | C:0.52  **A:0.48** | | **C:0.34**  A:0.66 | | **C:0.13**  A:0.87 | < 10^-6 |
|  | *c50_est:824A>G* | Non-Synonymous | A:0.51  **G:0.49** | | A:0.52  **G:0.48** | | **A:0.26**  G:0.74 | | **A:0.10**  G:0.90 | < 10^-6 |
| *Cytochrome P450* | *cyp:188C>T* | Synonymous | C:0.57  **T:0.43** | | **C:0.3**  T:0.7 | | C:0.957  **T:0.043** | | C:0.77  **T:0.23** | < 10^-6 |
|  | *cyp:263A>G* | Synonymous | A:0.57  **G:0.43** | **A:0.29**  G:0.71 | | A:0.943  **G:0.057** | | A:0.76  **G:0.24** | | < 10^-6 |
| *Endocuticle structural glycoprotein (SgAbd-8)* | *c480_est:926G>A* | Synonymous | G:0.53  **A:0.47** | G:0.55  **A:0.45** | | **G:0.17**  A:0.83 | | **G:0.009**  A:0.991 | | < 10^-6 |
|  | *c480_est:1051G>A* | Synonymous | G:0.53  **A:0.47** | G:0.55  **A:0.45** | | **G:0.17**  A:0.83 | | **G:0.009**  A:0.991 | | < 10^-6 |
| *Heat shock 70kDa protein* | *hsp_4:106G>A* | Synonymous | A:0.51  **G:0.49** | A:0.74  **G:0.26** | | **A:0.44**  G:0.56 | | **A:0.4**  G:0.6 | | < 10^-6 |
| *Vitellin-degrading protease precursor* | *c177_est:181A>G* | Synonymous | A:0.944  **G:0.056** | A:0.988  **G:0.012** | | A:0.61  **G:0.39** | | A:0.76  **G:0.24** | | < 10^-6 |
| *Glucose-6-phosphate 1-dehydrogenase* | *g6p1d:239C>T* | Non-synonymous | **C:0.23**  A:0.77 | **C:0.18**  A:0.82 | | C:0.57  **A:0.43** | | C:0.975  **A:0.025** | | < 10^-6 |
| *Serine proteinase-like protein* | *c3917_est:386A>C* | Synonymous | A:0.76  **C:0.24** | A:0.81  **C:0.19** | | A:0.86  **C:0.14** | | A:1  **C:0** | | < 10^-6 |

(*) Data from the 2007 pilot study
