# Supplementary material for: Genetic effects on life-history traits in the Glanville fritillary butterfly
Source: PeerJ. 2017 May 25;5:e3371. doi: 10.7717/peerj.3371 (PMC5446771; doi:10.7717/peerj.3371)
Supplement: Supplemental Information 16 — Each Pgi locus was genotyped twice. HW, Hardy Weinberg equilibrium; MAF, Minimum Allele Frequency; Gen, Genotyping; Homoz, Homozygote. [file peerj-05-3371-s016.docx]

**Table S1: Number of male and female individuals genotyped for each population (Åland, Gotland, Öland, Saaremaa, and Uppland)** in the main experiment (2009) and the pilot experiment (2007), and number of individuals included in each PCA1 (larval and pupal traits only), PCA_M_ (male adult traits only) and PCA_F_ (female adult traits only).

**Table S2: Pearson correlation matrices for the larval and pupal traits, the adult male traits and the adult females traits.** Values are Pearson correlative values (*R*) and asterisks denote significance at the 0.05 (*), 0.01 (**) and 0.001 (***) levels. The Bonferroni alpha values were corrected for 27, 15 and 91 correlations, respectively.

**Table S3: Eigen values, cumulative proportion of variance value and component loadings for the four first principal components of the principal component analysis performed on the larval and pupal traits.** Highest values from each PCA appear in bold for easier visualization of the results.

**Table S4: Eigen values, cumulative proportion of variance value and component loadings for the three first principal components of the principal component analysis performed on the male adult traits.** Highest values from each PCA appear in bold for easier visualization of the results.

**Table S5: Eigen values, cumulative proportion of variance value and component loadings for the three first principal components of the principal component analysis performed on the female adult traits.** Highest values from each PCA appear in bold for easier visualization of the results.

**Table S6: Minimum allele frequency (MAF), and minor allele for the 49 SNPs genotyped**. Each *Pgi* locus was genotyped twice. SNPs tagged with “(f)” failed our quality criteria, while SNPs tagged with “(*)” were genotyped for the 2007 pilot study. HWE: Hardy Weinberg Equilibrium; MAF: Minimum Allele Frequency; Gen: Genotyping; Homoz: Homozygote. Åland (ÅL), Gotland (GO), Öland (ÖL), Saaremaa (SA), and Uppland (UP).

**Table S7: Details of statistical analyses showing significant results, including statistical models used for each trait, as well as the explanatory variables included in the models.** Models of inheritance are given within brackets as Dominant, Recessive or Additive. Both main effects and interactions are reported. “NS” stands for non-significant.

**Table S8: The 15 SNPs showing significant environment effect on allele frequency, with the type of substitution and allele frequencies.** The minimum allele frequency for each SNP is shown in bold to ease visualization of the results. Allele frequencies differences between environments were calculated using directed permutation tests (1e+6 random permutations). The *p-*values give the posterior probability of the hypothesis that there is no difference between the environment types.
